# Rationale and methods for a cross-sectional study of mental health and wellbeing following river flooding in rural Australia, using a community-academic partnership approach

**DOI:** 10.1186/s12889-019-7501-y

**Published:** 2019-09-11

**Authors:** J. M. Longman, J. Bennett-Levy, V. Matthews, H. L. Berry, M. E. Passey, M. Rolfe, G. G. Morgan, M. Braddon, R. Bailie

**Affiliations:** 1 The University of Sydney, University Centre for Rural Health, 61 Uralba Street, Lismore, New South Wales 2480 Australia; 2 Sydney School of Public Health, Edward Ford Building, University of Sydney, Sydney, New South Wales 2006 Australia

**Keywords:** Floods, Disaster management, Mental health, Vulnerable populations, Climate change

## Abstract

**Background:**

Climate change is associated with greater frequency, duration, intensity and unpredictability of certain weather-related events, including floods. Floods harm mental health. There is limited understanding of the mental health and well-being effects from river flooding, particularly over the longer term and in rural contexts. This paper describes the rationale, aims, objectives, study design and socio-demographic characteristics of the sample for a study measuring associations between flood experience and mental health and wellbeing of residents (particularly those most likely to be negatively impacted and hard to reach) in rural NSW Australia 6 months following a devastating flood in 2017. To our knowledge, the study is the first of its kind within Australia in a rural community and is an important initiative given the likelihood of an increasing frequency of severe flooding in Australia given climate change.

**Methods:**

A conceptual framework (*The Flood Impact Framework*) drawing on social ecological approaches was developed by the research team. It was based on the literature and feedback from the community. The Framework describes putative relationships between flood exposure and mental health and wellbeing outcomes. Within a community-academic partnership approach, a cross-sectional survey was then undertaken to quantify and further explore these relationships.

**Results:**

The cross-sectional survey was conducted online (including on mobile phone) and on paper between September and November 2017 and recruited 2530 respondents. Of those, 2180 provided complete demographic data, among whom 69% were women, 91% were aged 25–74, 4% identified as Aboriginal and/or Torres Strait Islander, 9% were farmers and 33% were business owners.

**Conclusions:**

The study recruited a wide range of respondents and the partnership facilitated the community’s engagement with the design and implementation of the study. The study will provide a basis for a follow-up study, that will aim to improve the understanding of mental health and wellbeing effects over the longer term. It will provide an important and original contribution to understanding river flooding and mental health in rural Australia, a topic that will grow in importance in the context of human-induced climate change, and identify critical opportunities to strengthen services, emergency planning and resilience to future flooding.

## Background

In late March/early April 2017 extreme rainfall from ex-Tropical Cyclone Debbie resulted in river flooding in the Northern Rivers, a rural area on the north coast of New South Wales, Australia with a sub-tropical climate. Almost all of the rain fell within 24 h and flooded many regions of the Northern Rivers inundating the major population towns of Lismore and Murwillumbah, with extensive damage to housing and infrastructure. For many areas it was as severe as the worst flood on record (1974).

In 2015, the economic cost of weather-related and other natural disasters in Australia was estimated to exceed $9 billion with the social cost (e.g. impact on health and wellbeing, education, employment) contributing an equivalent or larger component than physical infrastructure costs [1]. This annual cost is estimated to double by 2030, not counting the potential impacts of climate change [1]. Floods are the most expensive weather-related event experienced in Australia [2].

Analysis of global flood data and associated population impact from 1975 to 2016 showed a significant increase in flood-affected population and mean annual flood-induced mortality in Australia [3]. Based on the output of a number of climate models, an increase in the frequency of floods is likely along the east coast of Australia [4].

There are two broad categories of floods: coastal floods caused by high tides and storm surges; and fluvial (river) flooding caused by heavy rainfall in river catchment areas [5]. River floods are the most common flood disasters globally [3].

### Flooding and mental health

The related constructs of mental health and wellbeing (the subjective experience of affect and life satisfaction, psychological functioning and self-realisation [6]) influence individuals’ ability to cope with everyday life stresses, relationships with others, working productively, contributing to community and fulfilling one’s potential [6, 7]. Although damage from flooding to the built and natural environment and, in some instances, damage to physical health is immediately evident, floods can also harm mental health and wellbeing contemporaneously and subsequently. These harms can be substantial. For example, in the UK, mental health problems have been estimated to account for 80% of all Disability Adjusted Life Years attributable to floods [8]. While the most immediate effects of flooding (injuries, infections, chemical hazards, and disruption to health and social services) are well documented, the mental health and wellbeing effects of river flooding, particularly in rural areas and over time, are less well understood [2, 9].

### Study aims and objectives

**Fig. 1:**
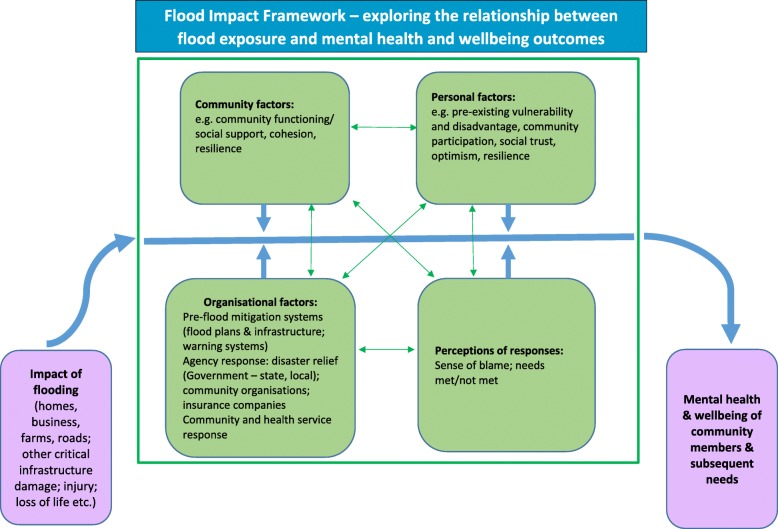
Flood Impact Framework – exploring the relationship between flood exposure and mental health and wellbeing

This study therefore aimed to measure mental health and wellbeing 6 months following the flood in rural NSW, and explore the association between flood exposure and mental health and wellbeing to quantify and better understand the associations in relation to a proposed Flood Impact Framework (Fig. 1), in order to inform current and future disaster support and mental health service provision. The specific objectives of the study were to:

1. describe the extent of the impact of the April 2017 flood on the physical environment of communities in the Northern Rivers’ Region
2. explore the associations between mental health and wellbeing and:
  - the nature and extent of exposure to flooding
  - perceptions of the adequacy of pre-flood warning systems, plans and mitigation infrastructure and the subsequent disaster relief service response
  - levels of community and personal resilience
3. conduct subgroup analyses of the association between flooding and mental health and wellbeing of the following key interest groups who are disproportionately vulnerable to the effects of weather-related events: respondents living in disadvantage (indicated by receipt of government income support); business owners; farmers; respondents identifying as Aboriginal and/or Torres Strait Islander; respondents 75 years and older; and the young (16–25 years).

### Ethical approval

The study, sub-study and related study were approved by the University of Sydney Human Research Ethics Committee (reference-2017/589) and the Aboriginal Health and Medical Research Council Human Research Ethics Committee (reference-1294/17). Potential respondents were advised that completing the questionnaire would be taken to mean their consent to participate in the study.

### Community-academic partnership

A community-academic partnership [10] was integral to the design, development and implementation of the study, in particular to recruiting participants to the study [11]. The partnership developed over time, beginning in late April 2017 just a few weeks after the flood, and is ongoing. It has taken many forms including recruiting new staff from the community into the research team and establishing two Community Advisory Groups (CAGs). Details are expanded below under ‘Study Design’. Partnerships between community and researchers have been described as central to addressing the gap between evidence and practice [11, 12] and the benefits of these partnerships include ensuring the relevance of research questions and designing studies to be of direct use to the community [11].

### The flood impact framework

A conceptual framework, the ‘Flood Impact Framework’ (Fig. 1) was developed by the academic research team and was based on prior empirical research on flood and other natural disasters. We presented the draft Framework to the community (see Fig. 3 – timeline) for discussion, adjustment and eventual agreement based on their expertise and experience. The discussion was primarily around capturing the factors which might contribute to or mitigate the effects of being exposed to a flood and mental health and wellbeing outcomes. Community members advised us in the context of their community membership as well as their work/voluntary roles. The Framework provided a starting point to articulate potential relationships between flood exposure and mental health and wellbeing outcomes (purple boxes), taking into account other contributing factors (green boxes), and helped guide the choice of measures for the questionnaire. The key path of interest in the Framework was between exposure to the flood and mental health and wellbeing outcomes. The factors identified (the four green boxes) act as potential mediators of that path. All factors interact. The utility of the Framework will be reviewed in the light of findings from our research described here including the survey results, and refined accordingly.

Drawing on social ecological models which recognize multi-level influences on health outcomes [13–15], the Flood Impact Framework suggests that a combination of personal, community, organisational factors, a person’s response to these factors, and degree of flood impact predict mental health and wellbeing outcomes. These factors can be proximal, intermediate and distal and may interact directly or indirectly [16]. The Framework is focused on the social aspects of social ecological models (although we acknowledge the potential health implications of changes in biophysical/living systems). The Framework reflects assumptions that community, personal, organisational and response factors mediate the impacts of flooding on mental health and wellbeing, and that these mediating factors may directly influence the impact of each other; for instance, a belated agency response in a flooded area may directly affect the community’s capacity to provide support for one another, which in turn may affect individuals’ trust and optimism, and whether their needs were met or not met by agencies and their community.

A prior example of the use of a social ecological framework to promote recovery after a natural disaster is provided by The Joint Centre for Disaster Research following the 2010–11 New Zealand earthquakes [17]. Paralleling the Flood Impact Framework, these authors identified individual, community, and societal/agency factors as key contributors to resilience/adaptive capacity. What the Flood Impact Framework adds to the New Zealand framework is a specific focus on the direct and indirect impacts of flooding, and whether or not individual needs are met by community, organisational and personal resources.

Although the Flood Impact Framework was developed prior to the publication of a recent systematic mapping study of the long-term physical and psychological health impacts of flooding [18], the factors in the Framework closely match the factors outlined in this mapping study. What the present Framework adds to the systematic mapping study [18] is hypothesis-generating capacity by positing putative links between different factors in the framework. Similarly, the Flood Impact Framework reflects a number of the factors impacting on mental health identified in a recently published UK study of wellbeing after floods (e.g. dislocation from home, community factors, public policy, emergency responses, perception of responses, etc.) [19].

The key elements of the Framework (impact of flooding; community, personal and organisational factors, and perceptions of organisational responses) are outlined below and have previously been associated with negative mental health and wellbeing outcomes following extreme weather-related events.

### Impact of flooding

The direct and indirect impact of flooding, such as house inundation [20–22], displacement [23], businesses flooded [24] and/or disrupted access to services [21, 25] have been associated with elevated negative mental health outcomes compared to unexposed groups.

### Community factors

Community cohesion [26–29], resilience [2, 30, 31] and participation [26, 27, 32] in the form of informal social connectedness (such as having contact with friends, family and neighbours) [18, 27, 33], and civic engagement (such as participating in organised community activities) [2, 32] appear to play an important role, influencing the link between extreme weather-related events and mental health outcomes. Community cohesion may often mitigate negative mental health impacts, but in some cases community divisions or inappropriate volunteer support can heighten negative impacts [19].

### Personal factors

Personal factors such as pre-existing vulnerability and disadvantage [21, 30, 34], previous flood experience [9], and personal resilience [35] similarly contribute to the combination of factors which predict mental health and wellbeing outcomes. For instance, people who are socio-economically disadvantaged are more likely to live in flood-prone areas [36] and tend to have fewer resources to recover from its impacts [21, 34]. Those living in rural and remote areas [37] and older adults [38] are also more vulnerable to the effects of flooding.

### Organisational factors

Finally, organisational factors contribute; for example, pre-flood mitigation systems, and warning systems [2], the response of Federal, State and local governments, community organisations and insurance companies [39, 40] all affect the mental health impact of experiencing a flood. In particular, lack of support from insurance companies has been extensively implicated in ongoing mental health problems [39, 41, 42]. The immediate and ongoing response of health and community services to weather-related events has been shown to be an important contributing factor to mental health and wellbeing and to recovery following severe weather-related events [43, 44]. ln the English flood study described previously, perceived lack of evacuation warning was associated with greater depression and post-traumatic stress disorder (PTSD) [23].

### Perceptions of organisational responses including blame

The community raised a number of issues around warning about the flood including that it was: not received by some, too late for some, too early for some (particularly business owners), inaccessible for some, gave inconsistent information, and/or was not sufficiently detailed. This was discussed with the community including at CAG meetings. As highlighted in other research [19], the Framework therefore also included perceptions of organisational responses, including blame [9] (to explore whether blame for perceived failures in Government’s or agencies’ responses might contribute to mental health outcomes [40, 45]).

This paper describes the rationale, aims, objectives, study design and socio-demographic characteristics of the sample for a study measuring associations between flood experience and mental health and wellbeing of residents in rural NSW Australia 6 months following devastating flooding in 2017. The results of the study (the cross-sectional survey) and the related study (flood mapping study) will be published separately to this paper.

## Methods

### Study location

**Fig. 2:**
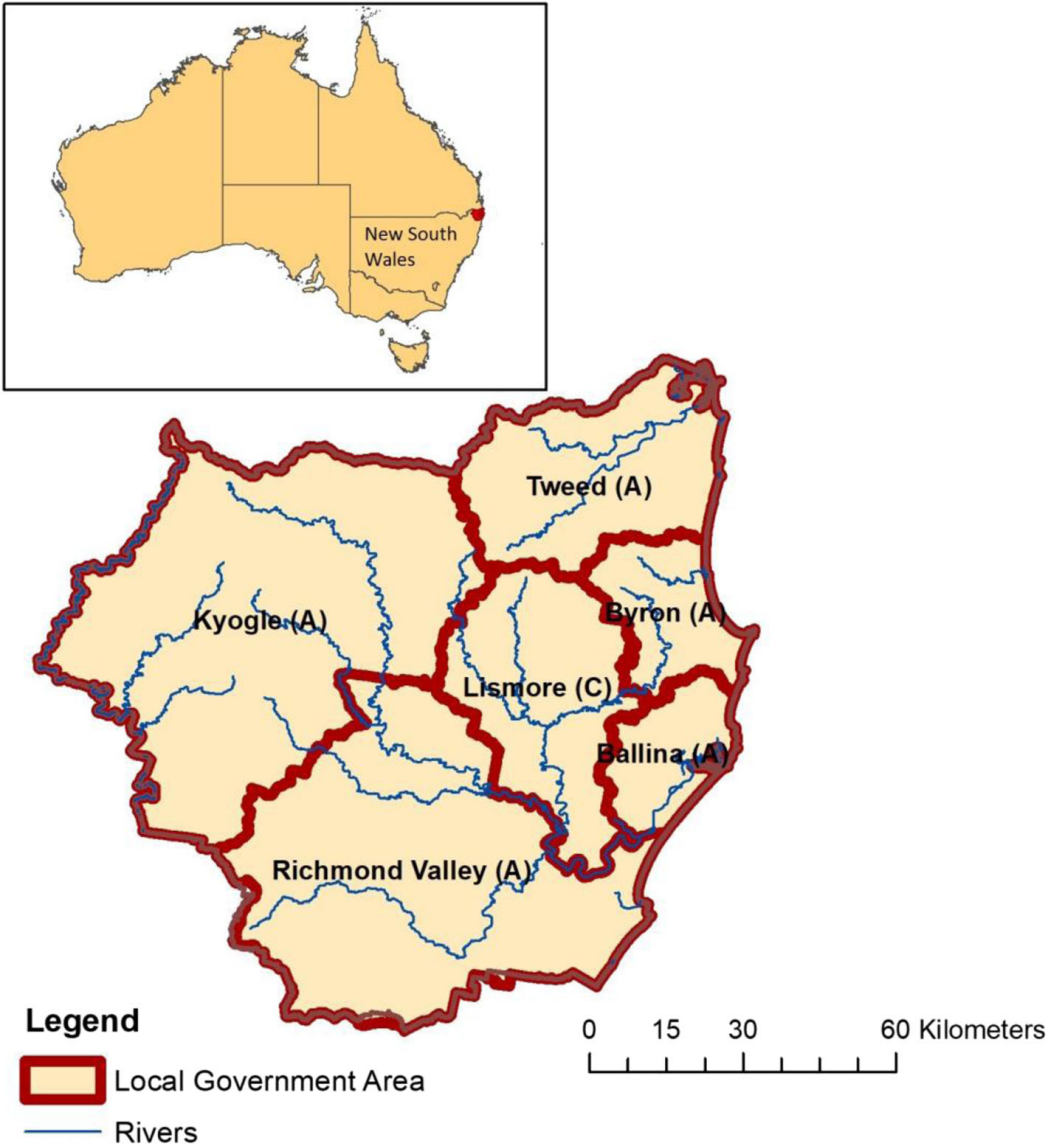
Study location

Six Local Government Areas (LGAs) within the Northern Rivers region were included: Ballina Shire, Tweed Shire, Richmond Valley, Kyogle, Byron Shire and Lismore City (Fig. 2). The total estimated residential population of these LGAs was 239,604 in 2016 [46]. From the Australian Bureau of Statistics (ABS) 2016 census estimates (5 year age groups), 82% of this population was 15 and over [47]. The region has higher proportions of older people and Aboriginal people compared to state averages and has experienced recent high population growth driven by coastal migration and counter-urbanisation. The region includes many areas of socio-economic disadvantage (in 2016 27% of the population was living in the lowest quintile of socio-economic disadvantage, with a range of 6–58% across different locations) [48]. The region is a known hotspot for weather-related extreme events, particularly flooding [36].

### Study design

**Fig. 3:**
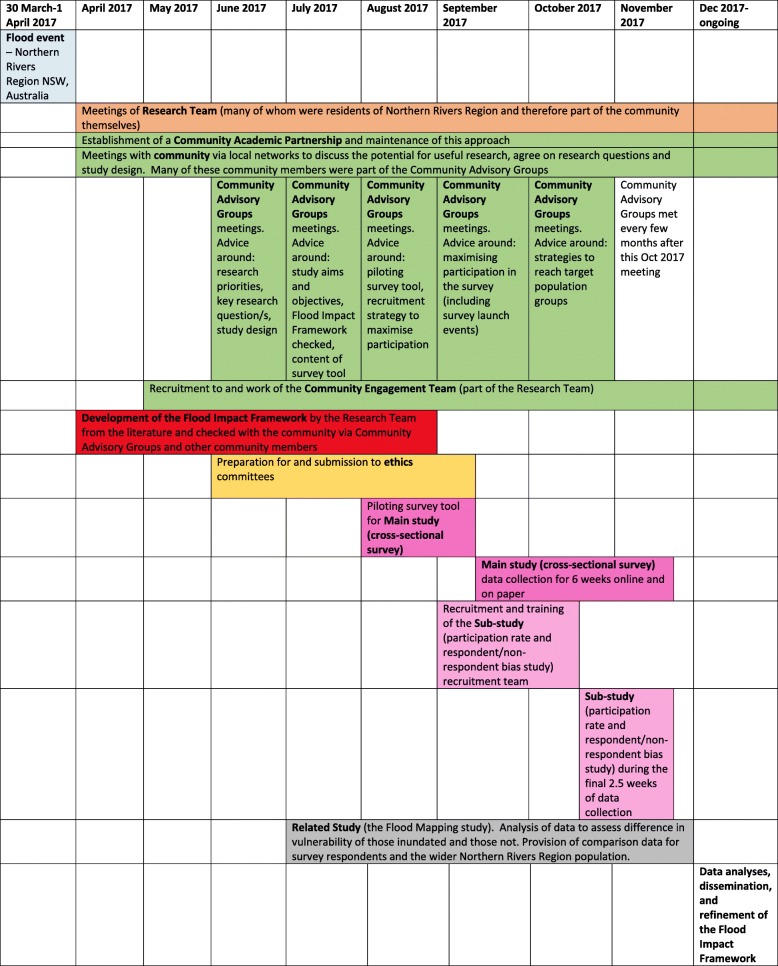
Timeline – Main study, sub study and related study of mental health and wellbeing following river flooding

The whole study was underpinned by a community-academic partnership approach and consisted of a Main study (which collected data using a cross-sectional survey) with one sub-study (measuring the participation rate and respondent/non-respondent bias in the Main study) and one related study (a flood mapping study). Fig. 3 shows a timeline of how these elements of the study fitted together.

### Main study

The Main study was a cross-sectional survey. The survey was made available online, on mobile phone and in paper form between September and November 2017.

#### Recruitment methodology

The aim of recruitment was to reach people living in the Northern Rivers region at the time of the 2017 flood who experienced damage to any of five physical locations or structures: suburb; non-liveable areas of their home (e.g., garden shed, garage); liveable areas of their home (e.g., bedrooms); income-producing property (business/farm, if applicable); and the home of a significant other, as well as those who were not exposed (no surrounding infrastructure damage, evacuation or displacement). As some of the key interest groups are difficult to reach (e.g., people living with disadvantage), purposive sampling utilising a snowball technique recruiting respondents via personal and local organisational networks and encouraging respondents to raise awareness of the survey with friends, family and colleagues was conducted. As the study did not aim to assess population prevalence of flood exposure or mental health outcomes but rather to quantify relationships between flood impact and mental health risk the sample was not randomly selected, but was purposively recruited [49]; the focus was to ensure adequate numbers of respondents from key interest groups to enable analysis of exposure and outcome for these groups.

Community-academic partnership was key to the study design and implementation, particularly in facilitating recruitment to the Main study and in providing support for respondents completing the questionnaire. The partnership with community took many forms including recruiting two new staff members from the community into the research team with a focused community engagement role; one with an impressive track record in local TV/radio and print media journalism and the other, one of three women who were the driving force behind the establishment of an inspiring self-organising group (*Helping Hands*) connecting hundreds of volunteers with locals requiring support following the flood. The leader of, and the five members of the recruitment team for the Sub-study (see *Sub-study* section below and Fig. 3) were also highly networked, experienced, and well known members of the community.

Two Community Advisory Groups (CAGs) were also established one in Lismore and one in Murwillumbah. The CAGs included representation from around 60 local health and community organisations, business groups and state and local government authorities, met frequently and provided critical advice on the research questions, study design, recruitment strategies, questionnaire content (including piloting), analysis priorities and dissemination strategies.

The community-academic partnership was initiated by academic researchers. Members of the partnership were not funded to participate in the partnership, their commitment to contribute (either in one-to-one meetings by phone or face to face, or by being a member of one of the CAGs and attending CAG meetings) stemmed from a commitment to the community, grounded in the shared experience of the flood. The partnership was large in comparison to other studies [10] and was made up of NGOs, community organisations, local government, service providers, members of the public, the business community and others, many of whom were members of the CAGs. The CAGs had agreed Terms of Reference which helped to clarify purpose and roles. The nature of the community-academic partnership was characterised by a goal (to successfully complete the research and disseminate the findings so that they could be used to inform improved support for the community before, during and after flooding), relevant to the community, and involved community members as well as academics. As such the community-academic partnership was congruent with the conceptual definition of community-academic partnerships by Drahota et al. [10] Description of the timeline for the partnership is provided in Fig. 3.

Initial invitation to participate in the survey (most commonly by email) was via social and organisational networks of community organisations, the CAGs, the local health service, local government authorities and business/farming groups. For example, the local health service (one of the largest employers in the region) who were part of the CAGs sent an email to their all-staff list inviting participation and including a link to the questionnaire online and instructions about how to access a paper version of the questionnaire if preferred. This approach was supplemented by an extensive local media (print and broadcast) advertising campaign, fliers and posters (which included a QR code - a 2-dimensional bar code that enabled potential respondents to access the survey website easily using their mobile phones). The posters and leaflets along with paper surveys (with franked return envelopes) were placed in central community locations such as council offices, coffee shops, in every library and post office, and in shops belonging to charitable organisations such as Lifeline, Interrelate, St Vincent de Paul and the Salvation Army. Social media was also used intensively and strategically to raise awareness and invite potential respondents, including Twitter and a Facebook page incorporating short videos of key community members talking about the survey. The survey was launched at face-to-face media and community events marking 6 months since the flood. The team’s community engagement staff had high visibility in the community throughout the duration of the survey period by staffing a stall at farmers’ markets and attending a plethora of other community events. Appropriately skilled members of relevant organisations (e.g. Lifeline, a crisis support service) received information about the ethical aspects of conducting the survey and provided one-to-one support for survey completion. Door-to-door recruitment took place in Lismore and Murwillumbah.

When participation in the Main study was reviewed with the community via the CAGs at the halfway point a number of specific strategies were discussed, agreed and employed to maximise participation from men (promoting the survey via men-specific organisations such as the Men’s Shed, posting videos on the project’s Facebook page of men talking about completing the questionnaire), older people (taking paper copies of the questionnaire and advertising material to residential aged care facilities) and people aged 16–25 years (promoting through networks of youth workers and Facebook). A leaflet delivered by post to residents in Lismore and Murwillumbah was also added at this point.

A lottery style draw of gift vouchers for $100 to spend in local businesses was offered to respondents who opted to put their details into the draw.

#### Questionnaire development

A draft questionnaire based on the conceptual Flood Impact Framework was developed by a leading expert in extreme weather-related events and their impacts on population mental health, the scientific team and in partnership with the community. Where possible, pre-existing validated measures and survey tools from previous flood research [22, 25, 34] (to facilitate comparison) were used. Where necessary, new measures were developed. The CAGs facilitated piloting the penultimate version of the questionnaire (which was then revised into the final version) by recruiting from their networks 30 volunteers from various socio-demographic backgrounds.

#### Questionnaire content

The final 58-item questionnaire covered: socio-demographic characteristics; the six flood exposures established a priori (suburb, non-liveable areas of their home, liveable areas of their home, business/farm and/or the home of a significant other flooded, plus not exposed to any of these evacuated or displaced); respondents’ experiences during the flood including evacuation and displacement; mental health items and items measuring individual and community resilience and social capital. Table 1 contains information on the main measures and their origins and how they relate to the Flood Impact Framework. In addition to these items, the questionnaire contained eight free-text opportunities inviting respondents to report their perspectives on their flood experience.

**Table 1:** Main questionnaire items, their origins and how they relate to the Flood Impact Framework (Fig. 1)

Item	Origin (*and scoring where relevant*)	Relationship to Flood Impact Framework
Socio-demographics variables: • Age • Gender • Indigenous status • Relationship status • Education level • Employment status • In receipt of income support • Farmer • Business owner	N/A	Personal characteristics, including those identifying key interest groups
Flood exposure (liveable area of home flooded; business flooded; non-liveable area of home flooded e.g. garage; suburb flooded; home of close friend or relative flooded; none of the above). Degree of flooding: water above your head height through entire home/property; water between knee and head height (more than 50 cm) through entire home/property; water below knee-height (about 1-50 cm) through entire home/property; water in some but not all areas of home/property	Derived from the Brief Weather Disaster Trauma Exposure and Impact Screen [9] and the English National Cohort Study on Flooding & Health [22].	Impact of flooding
Evacuation: • Did you have to evacuate your home/business? • How much warning did you get?	Derived from the English National Cohort Study on Flooding & Health [22].	Impact of flooding Pre-flood mitigation systems: Warning systems
Displacement: • Because of the flood did you have to live elsewhere?	Derived from the Brief Weather Disaster Trauma Exposure and Impact Screen [9] English National Cohort Study on Flooding & Health [22].	Impact of flooding
Support at the time of the flood: • Did support requested from Govt/Community organisations/insurance/emergency services/volunteers meet your needs?	Bespoke measure developed for the Flood Impact Framework	Agency response: disaster relief Perceptions of responses Community & health service response: mental health & wellbeing needs
Blame: • Are Govt/Community organisations/insurance/emergency services/volunteers to blame for distress?	Bespoke measure developed for the Flood Impact Framework	Perceptions of responses: sense of blame
Previous flood experience: • Have you ever been in heavy rain or floods in which your home, business, workplace or school was damaged?	Bespoke measure developed for the Flood Impact Framework	Previous flood exposure, cumulative flood exposure
Post-traumatic growth: • Have the severe rain and flood resulted in you being able to make any positive changes in your life?	Bespoke measure developed for the Flood Impact Framework	Personal factors
Individual and community resilience: • Personal social capital – community participation	Australian Community Participation Questionnaire [50]. *Seven point agree/disagree scale*	Community factors Personal factors
• Community functioning	A measure of ‘social’ (or ‘generalised’) trust from Berry et al. 2003 [51], a question from the CRACE study [52] and a bespoke measure for the Flood Impact Framework	Community factors
• Personal social cohesion - connection, sense of belonging & support	Two sub-scales from the Interpersonal Support Evaluation List (Cohen et al. 1985 [53]) Berry 2008 [54] *Seven point agree/disagree scale*	Community factors Personal factors
• Social trust	Adapted by Berry, 2008 [55] from the Organisational Trust Inventory (OTI) (Cummings & Bromiley,1996 [56]) and the World Values Survey (Inglehart et al., 2000 [57]) *Seven point agree/disagree scale.*	Personal factors
• Generalised reciprocity	Adapted by Berry, 2008 [55] from the World Values Survey (Inglehart et al., 2000 [57]) *Seven point agree/ disagree scale*	Personal factors
• Trait optimism	Adapted from the Life Orientation Test – Revised (LOT-R) (Scheirer et al. 1994) [58] *Seven point agree/disagree scale*	Personal factors
Mental health and wellbeing outcome measures: • Flood-specific - Still distressed about the flood	Brief Weather Disaster Trauma Exposure and Impact Screen [9] “*Are you still currently distressed about what happened during the flood?*” *Yes/No*	Mental health & wellbeing of community members and subsequent needs
• Flood-specific - Post-Traumatic Stress Disorder (about the flood)	Post-Traumatic Stress Disorder Checklist (PCL-6) [59]. A list of complaints that people sometimes express after extreme rain and flooding. *Cut-point for probable diagnosis was ≥ 14* [59]	Mental health & wellbeing of community members and subsequent needs
• Not flood-specific - Depression	Patient Health Questionnaire (PHQ-2) [60]. *Cut-point for probable diagnosis was ≥ 3* [60]	Mental health & wellbeing of community members and subsequent needs
• Not flood-specific - Anxiety	Generalised Anxiety Disorder scale (GAD-2) [61]. *Cut point for probable diagnosis was ≥ 3* [61]	Mental health & wellbeing of community members and subsequent needs
• Not flood-specific - Suicidal ideation	A single suicidal ideation item from the Screening Tool for Assessing Risk of Suicide [62] *Yes/No*	Mental health & wellbeing of community members and subsequent needs

As the questionnaire was rather long, respondents were offered the choice between completing a shorter (15 min) or longer (25 min) version. The shorter version (the first part) contained socio-demographic variables, flood experience items, mental health measures and a post-traumatic growth measure. The longer version included all of the above as well as personal and community resilience measures.

Consistent with previous research, a high level of community distress following the flood was anticipated [2, 38, 63]. Caring for respondents and minimising the risk of harm to their mental health was, therefore, a core component of the questionnaire design. For example, the number of difficult items was minimised, more difficult items (e.g., items about suicide) were located after or before less difficult items, free-text opportunities were included, any distress caused by completing the questionnaire was acknowledged and apologised for in the introductory material, and contact information for counselling and support services featured prominently throughout.

At the end of the survey (short and full versions) respondents were asked if they would be willing to be contacted in future to participate in further research about the flood. The online survey was generated using Qualtrics software (version Sept-Nov 2017, Qualtrics Provo Utah).

#### Planned data analyses

The dataset for the Main study has been cleaned and an initial descriptive analysis including means and standard deviations, frequencies and proportions for all social and mental health and wellbeing variables undertaken (Matthews V, Longman JM, Berry HL, Passey ME, Bennett-Levy J, Morgan G, et al.: Mental health six months after extensive flooding from cyclone Debbie in rural Australia: a cross-sectional analysis through an equity lens, submitted). These statistics will be calculated separately for the sample as a whole, for the six exposure groups and the key interest groups. Mental health and wellbeing outcomes across each of the exposure and key interest groups will be examined. Respondents reporting none of the exposures will form a control group for comparison to groups which reported one or more exposures. Analysis of proposed protective factors for mental health and wellbeing (such as community resilience) between the different flood-exposure groups will be undertaken. A broad range of inferential statistical procedures will be employed to describe relationships between exposure and outcome variables and associations with other factors according to the proposed Flood Impact Framework. These may include calculation of correlation coefficients, analyses of variance, hierarchical and logistic regression analyses, cluster analyses and multi-level and structural modelling. The necessary data have been collected to adjust for a wide variety of factors known to predict psychiatric morbidity. Analyses will assist in evaluating the plausibility of the proposed Flood Impact Framework and in improving both the Framework and future study design.

Free text data will be analysed deductively using a content analysis approach following Elo et al. [64]

### Sub-study - The participation rate and respondent/non-respondent bias sub-study

A randomised stratified cluster sample sub-study was conducted to examine participation rate and respondent/non-respondent bias within the Main study sample in the flooded areas of the two major population centres impacted by the flooding (Lismore and Murwillumbah). The sub-study aimed to: assess participation rates achieved through the recruitment strategies employed in the Main study, and thus provide evidence of the effectiveness of these strategies in the most flooded areas; determine characteristics of people in the sample who specifically declined to participate in the survey, for the purpose of assessing respondent/non-respondent bias; and to maximise recruitment of people in areas most inundated by the floods.

The sub-study involved door-to-door recruitment within clearly defined areas in Lismore and Murwillumbah based on ABS 2016 census mesh blocks (around 100 dwellings per block, and the unit of random selection), stratified by land use pattern (residential, primary production or commercial) and exposure classification (from local council maps indicating that the land was flooded or not flooded). Mesh blocks that were flooded were weighted such that they had twice the probability of selection. Three attempts to collect data from every household within each selected mesh block were made. Within households, all residents ≥16 years old were eligible for inclusion in the sub-study and invited to participate. The door-to-door recruitment was undertaken by local skilled and trained recruiters who also assisted people in completing the questionnaire on computer tablets or on paper if required. Within each household, data were collected on the number of residents ≥16 years who were living in the study area at the time of the flood, the number of residents responding to the sub-study, and for each of these respondents, their age, gender, whether or not they had heard about the flood survey (Main study), whether they had completed it and whether they were willing to do so now. The sub-study was undertaken during the final two and a half weeks of recruitment for the Main study (see Fig. 3), and took twelve working days.

### Related study - the flood mapping study

The aim of this related study was to compare socio-demographic and selected health characteristics of Northern Rivers residents who lived within areas inundated by floodwater from the 2017 flood with those who lived in areas that were not inundated, in order to assess difference in vulnerability between those inundated and not. The study used flood maps (the “flood footprint” i.e. where the flood water was located) provided by the NSW Office of Environment and Heritage together with flood maps from local councils to compare characteristics of residents who lived in the flood footprint with residents of the wider Northern Rivers community. This included:1) describing the population-level socio-demographic and health characteristics of flood footprint residents by overlaying information from the ABS 2016 Population Census [65] and the large cohort study [66] with flood maps, and comparing the flood footprint residents with the wider Northern Rivers population; and 2) comparing the socio-demographic characteristics of the survey respondents with the Northern Rivers population. Findings from this study are currently being prepared for publication and will be disseminated extensively as part of the community-academic partnership.

## Results

Over 2500 people participated in the survey, with three-quarters participating online and the vast majority (89%) completing both sections of the questionnaire. Some 2180 (86%) respondents provided full demographic data (Table 2).

**Table 2:** demographic characteristics, flood exposure and mode of participation of respondents

			*n* (%)	
Number and mode of respondents:				
Total respondents			2530 (100)	
Respondents online			1907 (75)	
Respondents on paper			623 (25)	
Respondents completing both parts of questionnaire (1 and 2)			2251 (89)	
*Total respondents providing full socio-demographic data*			*2180 (86)*	
	Online n (within row %)	On paper n (within row %)	*n* (% of 2180)	Population across the 6 local government areas *n* (%)
Total population across 6 local government areas				239,604
Socio-demographic characteristics (*n* = 2180)				
Women	1191 (79)	309 (21)	1500 (69)	123,343 (51)
Men	458 (67)	222 (33)	680 (31)	116,261 (49)
Age 16–24	102 (85)	18 (15)	120 (6)	24,367 (10)^a^
Age 25–74	1517 (76)	468 (24)	1985 (91)	149,566 (62)
Age 75+	30 (40)	45 (60)	75 (3)	24,592 (10)
Identified as Aboriginal and/or Torres Strait Islander	58 (75)	19 (25)	77 (4)	9739 (4)
Farmer or farm worker	144 (76)	45 (24)	189 (9)	4581 (5)
Business owner	502 (70)	212 (30)	714 (33)	Not available
Single (vs in a relationship, e.g., married)	497 (71)	207 (29)	704 (32)	73,240 (43)
Has a university degree	807 (84)	150 (16)	957 (44)	27,966 (14)
In paid employment (full or part-time)	1237 (82)	274 (18)	1511 (69)	96,421 (49)
In receipt of Government income support	428 (63)	248 (37)	676 (31)	69,389 (18)
Flood exposure groups (*n* = 2180)				
Suburb flooded	1224 (74)	435 (26)	1659 (76)	
Non-liveable areas of their home flooded	761 (74)	274 (26)	1035 (48)	
Liveable areas of their home flooded	306 (67)	154 (33)	460 (21)	
Business/farm flooded	268 (73)	97 (27)	365 (17)	
Home of a significant other flooded	1065 (77)	315 (23)	1380 (63)	
Not exposed to any of the above	153 (77)	45 (23)	198 (9)	
Future research (*n* = 2180)				
Willing to participate in further research (yes or possibly)	1219 (88)	163 (12)	1382 (63)	

^a^aged 15–24 available only

Approximately seven out of every ten respondents were women. Only 6 % of respondents were in the youngest age bracket (16–24) compared to the population of the study location (10%). Similarly, it was difficult to recruit older people (75+ years) into the survey who comprised only 3 % of respondents compared to 10% in the wider population. Farmers were over-represented in the sample (9% compared to 5% in the population), as were respondents in receipt of Government income support (31% compared to 18%), and one-third of respondents were business owners. Respondents identifying as Aboriginal and/or Torres Strait Islander Australians constituted 4% of the sample, matching the proportion in the local population. The large majority of respondents reported at least one flood exposure (91%) compared to those who did not (9%).

Compared to other respondents, older respondents were more likely to complete the paper rather than online questionnaire (60% of older respondents) as were those in receipt of income support (37% completed the paper questionnaire rather than online). These respondents were found to reside in the more disadvantaged parts of the region which also suffered the worst of the flooding (Matthews V, Longman JM, Berry HL, Passey ME, Bennett-Levy J, Morgan G, et al.: Mental health six months after extensive flooding from cyclone Debbie in rural Australia: a cross-sectional analysis through an equity lens, submitted), as in other studies [34, 67].

The door-to-door participation rate and respondent/non-respondent bias sub-study was conducted in 17 randomly selected mesh blocks in the two main towns (Lismore and Murwillumbah), ten of which were in the flooded areas. The mesh blocks contained an estimated 1494 individuals in 903 residences. Of these, 1062 individuals and 663 residences were in the flooded areas (73 and 71% respectively). Data were collected from 713 individuals in 399 residences, 48% of the estimated resident population. Rates of awareness of the survey were similar within and outside the flooded areas (48 and 52%). The participation rate (individuals who had completed the survey prior to being door-knocked) was 4.9% from individuals who lived in the flooded areas and 5.0% from those outside these areas. Women were over-represented in the individuals who had already completed the questionnaire (69%). Individuals who had not completed the questionnaire were asked if they were willing to do it. A total of 110 declined (17%), the majority of whom (62%) did not live in the flooded areas, and 537 agreed.

## Discussion

Using a cross-sectional survey, in conjunction with a community-academic partnership approach, this study aimed to quantify relationships between river flood exposure and mental health and wellbeing in a rural region of NSW, Australia, focusing on key interest groups (older people, young people, farmers, business owners, Aboriginal and/or Torres Strait Islander people and those living with socio-economic disadvantage); and to further understand these relationships within the context of a proposed Flood Impact Framework. To our knowledge, the study is the first of its kind within Australia in a rural community and is an important initiative given the frequency of severe flooding and the likelihood that this will increase given climate change [2] (for example the latest IPCC report includes that no remaining Arctic sea ice is ten times more likely at 2 °C above pre-industrial temperature levels compared to 1.5 °C, which can lead to intense flooding [68]) and the substantial harms to mental health that flooding can bring [8].

The community-academic partnership led to a design that was oriented towards the priorities of the community and therefore resulted in community engagement with the study design and implementation, and in substantial community investment in the results. This approach offers the potential for research findings to influence on-going policy and service development as well as further research.

The Flood Impact Framework, like other social ecological approaches [13–15, 17], points towards the value of a systems-thinking approach [69]. It does this by incorporating the proposition that the mental health of individuals (in the context of climate change events) is profoundly influenced by a dynamic system of interacting factors. These include organisational and community capacity to respond effectively, social disadvantage (e.g. living in flood-prone areas, lack of access to insurance) and resource allocation. The factors identified are likely also to interact with biophysical/living systems though these are not a focus of the study. Typically, it is those people who are already significantly disadvantaged who are most impacted by weather-related events like floods [9, 70] and have access to the fewest resources in the face of climate change events [67]. Systems-thinking highlights the value of pitching interventions at multiple levels [69] simultaneously, for example at organisational and community levels as well as at individuals.

### Study strengths and limitations

There are a number of important methodological complexities associated with undertaking research of this nature. In order to address the key aims and objectives of the study, a non-probability, purposive sample with a snowball approach to recruitment was adopted. This was a pragmatic, appropriate, timely and affordable way to access key interest groups, some of which are known to be hard to engage in research [71]. This approach, while necessary, meant that the sample was not representative of the Northern Rivers population (as illustrated in Table 2) and therefore findings cannot be generalised to that population. Using a random sampling recruitment technique in a community the size of the Northern Rivers would likely not have resulted in sufficient power to compare between key interest groups e.g. flood affected farmers, or flood affected Aboriginal and/or Torres Strait Islander people. Whilst other studies of the association between flooding and mental health have employed more costly conventional approaches, including random-digit dialling and mailing out to households [22, 34], they have struggled with selecting appropriate sampling frames, low response rates and selection bias [2, 9, 22, 34, 72]. They have found it a challenge to engage difficult-to-reach populations such as people living in disadvantage [22], one of the key interest groups in this study.

The approach to recruitment in this study was successful in achieving the required sample size and in accessing a number of difficult-to-reach populations. It was also successful in raising awareness of the survey with around one half the population in the areas that were door-knocked. Given that the aim of the study was to identify relationships between exposure to the flood and mental health, rather than to assess prevalence (of exposure or outcomes) for the Northern Rivers population, the recruitment strategy focused on reaching potential respondents in the key interest groups rather than on securing a random and representative sample. Further, the door-to-door participation rate and respondent/non-respondent bias sub-study demonstrated that awareness of and participation rates in the survey were similar in the flooded and non-flooded areas targeted by the sub-study (Lismore and Murwillumbah) and participation was higher in these key target areas than in the overall Northern Rivers population.

The cross-sectional design of the study constrains the ability to make causal inferences. However, it supports the preliminary goals of exploring the plausibility of hypothesised associations between variables; testing new measures and concepts; and examining differences in the nature and extent of exposures and outcomes among key interest groups.

The mental health and wellbeing outcome measures used were based on validated clinical diagnostic tools rather than on asking respondents to recall receiving diagnoses (of depression, for example) in order to minimise potential self-reporting bias. Flooding, even widespread flooding, has extreme variation in impact, rendering it difficult to establish a denominator for population exposure and outcome measures. Self-reporting of exposure and outcome is, therefore, acceptable and has been widely and successfully used in other studies of the health impact of weather-related events [18, 22, 73–76].

As data were not gathered on respondents’ pre-existing mental health status, it remains uncertain how flood experiences may be related to mental health and wellbeing outcomes. However, two of the key mental health outcomes (*Still distressed about the flood*, and the measure of PTSD) were not about respondents’ general mental health but were specifically about mental health following the flood, and the analysis will control for other aspects of mental health and wellbeing as well as for factors known to be associated with poor mental health such as low socio-economic status.

## Conclusions

Presently, little is known about the association between river flooding and mental health and wellbeing outcomes in rural Australia. The study succeeded in recruiting a wide range of respondents, particularly in some of the key interest groups, and was committed to a community-academic partnership methodology. The partnership resulted in community engagement with the design and implementation and will assist with dissemination and use of findings. The study will provide a basis for a planned longitudinal cohort study to assess the short- (1–2 years) and medium-term (3–5 years) mental health and wellbeing outcomes of Northern Rivers’ communities affected by flood and their associated needs, improving understanding of mental health and wellbeing effects over time. It will facilitate exploration of the elements of the proposed Flood Impact Framework, improving understanding of the path that links exposure to river flooding and mental health and wellbeing outcomes following flooding. This will, in turn, enable exploration of critical opportunities to strengthen services, emergency planning and resilience to future flooding. In sum, this study will provide an important and original contribution to understanding river flooding and mental health in rural Australia, a topic that will grow in importance in the context of human-induced climate change.

## Data Availability

The datasets used and analysed during the current study are available from The University Centre for Rural Health on reasonable request.

## Abbreviations

ABS: Australian Bureau of Statistics
CAG: Community Advisory Group
LGA: Local Government Area
NSW: New South Wales (a State in Australia)
PTSD: Post-traumatic stress disorder
